# Profile and burden of the family caregiver: the caring experience in multiple sclerosis. An observational study

**DOI:** 10.1186/s40359-024-01678-w

**Published:** 2024-03-26

**Authors:** Michela Ponzio, Andrea Tacchino, Anna Verri, Mario Alberto Battaglia, Giampaolo Brichetto, Jessica Podda

**Affiliations:** 1 Scientific Research Area, Italian Multiple Sclerosis Foundation, Via Operai 40, 16149 Genoa, Italy; 2 Department of Physiopathology, Experimental Medicine and Public Health, University of Siena, Siena, Italy; 3 AISM Rehabilitation Service, Italian Multiple Sclerosis Society, Genoa, Italy

**Keywords:** Caregivers, Multiple sclerosis, Burden

## Abstract

**Background:**

The broad implications of caring for a family member with a chronic medical condition, such as MS, can lead caregivers to experience a high burden of care. The aim of the study was to describe profile of MS caregivers and their burden and to explore potential factors influencing this burden.

**Methods:**

200 family caregivers of a person with MS completed survey questionnaires across a cross-sectional study. Many information were collected: caregiver socio-demographic and health-related data, caregiving activities, knowledge of MS, coping strategies, mood, social support received and care recipient information. Caregiving burden was measured by the ZBI (Zarit Burden Interview). The extent to which the variables explained caregiver burden was analyzed using a hierarchical approach.

**Results:**

68% of the caregivers reported a perceived burden of care (ZBI score > 20). Our results show that physical and mental related-health variables are important predictive factors of the care burden, explaining much of the observed variance (40.9%).

**Conclusion:**

Family caregivers in MS continue to make up the shortfall produce by national health and welfare systems. We highlighted the importance of good physical and mental health in decreasing perceived burden. Working to alleviate psychological distress through mechanisms focus on reducing worries and perceived burden may be a valid approach.

## Introduction

An informal caregiver is any relative, partner, friend or neighbour who has a significant personal relationship with and provides a broad range of assistance to any individual who is not independent in caring for themself in some way [1]. Caregivers provide physical, practical and emotional support.

The broad economic, behavioural, functional, social, psychological, physical and medical ramifications of caring for a family member with a chronic medical condition can lead caregivers to experience a high burden of care [2], as seen in the case of multiple sclerosis (MS) [3]. Distinctive features of MS, namely its tendency to arise in young adulthood, its degenerative course, the absence of a definitive cure, and the variable disease course, unpredictable acute exacerbations, and varying clinical symptoms, can influence the caregiving experience. Many people with MS (PwMS), especially those with moderate to severe disability, require ongoing emotional, physical and practical support in order to manage the challenges of daily life and maintain their independence [4]. Most of this support comes from family members, who provide as much as 80% of home care to PwMS [5]. Carers provide assistance with basic personal hygiene and daily activities, provide emotional support, arrange for medical services and social assistance. As a result, they may experience high levels of chronic stress that can lead to deterioration of the carers’ health status, social life and well-being [6].

It is important to appreciate the role of informal caregivers and the extent to which they support healthcare systems around the world [7]. MS cost-of-illness studies showed the average annual value of informal caregiving in this setting to be €12,709 in 2015^8^, while in Italy, informal care generates 71.5% of the non-healthcare costs of MS [9].

The demands of caring for a person with MS can considerably impact the caregiver, resulting in diminished life satisfaction and high levels of stress, anxiety and depression [10]. An estimated 40% of caregivers of PwMS have clinical depression, and those who are also parents of PwMS appear to be particularly at risk [10]. Many of those caring for PwMS experience associated emotional strain [11].

Lower caregiver quality of life has been linked to insufficient support, financial difficulties, a lower level of education, and chronic illness in the caregiver [12]. Furthermore, many of those caring for PwMS feel under-informed about the disease, the available treatments, and how to manage various challenges, such as their own emotions and physical stress, as well as the care recipient’s safety in the home, possible cognitive deficits and difficult behaviour [13, 14].

To better support MS caregivers in their role, it is crucial to gain an understanding of their current experiences and needs so as to be able to find ways to decrease their perceived burden and ensure the well-being of both the caregiver and the care recipient. The strong family ties in Italian society and a specific attitude towards the use of family help contribute to making this objective relevant and urgent.

This study was conducted with two **aims**: (1) to provide a descriptive profile of Italian MS caregivers and their burden; (2) to explore potential factors influencing this burden.

## Method

### Sample

A cross-sectional study of family caregivers was carried out in Italy in 2021. In accordance with the definition provided by Buchanan et al. (2010), caregivers were included if they provided a variety of services, including personal care, homemaking, and assistance with daily activities, mobility and leisure activities, to a person with MS who was not fully independent in activities of daily living or in any other way at the time of the study [5].

Caregivers were enrolled through an Italian MS Society (AISM) network of psychologists (“*Rete Psicologi*”) between January and November 2021.

Data were collected using a questionnaire completed during an in-person or a telephone interview.

### Instruments

Using several scales (listed below), the questionnaire collected caregiver data, both socio-demographic (gender, age, level of education, employment status) and health-related (any medical conditions), as well as information on variables related to caregiving activities (type of support provided and intensity of caregiving activities). It also established each caregiver’s relationship with their care recipient and included questions about latter (gender, age, disease course and duration). Caregivers were also asked to complete specific scales exploring their perceived burden, knowledge of MS, coping strategies, mood, and perceptions regarding any social support received, as well as the care recipient’s level of disability and independence.

### Scales

The *Zarit Burden Interview (ZBI)* is the most widely-used instrument for measuring caregiver subjective burden [15], and we used the validated Italian version [16]. The ZBI contains 22 five-point Likert-style questions with reply options ranging from 0 “never” to 4, “nearly always”. The total score ranges from 0 to 88, with a higher score indicating a greater perceived care burden. The cut-off points are: 0 to 20 points = little or no burden; 21 to 40 = mild to moderate burden; 41 to 60 = moderate to severe burden; 61 to 88 = severe burden [17].

*Caregivers’ Knowledge of Multiple Sclerosis (CareKoMS)* measures what those caring for PwMS know about the disease. It has 21 items covering the following domains: etiopathogenesis, epidemiology, disease course, symptoms, and treatment. The items are multiple choice questions with only one correct answer. A higher number of correct answers indicates better knowledge of the disease [18].

*Coping Orientation to Problems Experienced-Nuova Versione Italiana (COPE-NVI-25)* evaluates the frequency with which a subject uses different coping strategies in difficult situations [19], and it can be considered a useful and psychometrically valid tool for measuring coping styles in Italian settings. It is comprised of 25 statements (items) divided into five dimensions: social support, positive attitude, problem solving, denial strategy, and religion. By responding to each statement (item), subjects indicate the frequency with which they apply various coping strategies: 1 = I don’t usually do this, 2 = I sometimes do this, 3 = I frequently do this, or 4 = I almost always do this. The score for each dimension is the sum of the values reported by the subject. However, since the scores for the different dimensions (coping strategies) are not all based on the same number of items, and are thus not comparable, a standardized total score was calculated for each dimension by dividing its total score by its number of items. This scale has already been used to test coping strategies in Italian caregivers of people with other diseases [20].

The *Hospital Anxiety and Depression Scale (HADS)* is a well-established instrument for identifying anxiety and depressive symptoms [21], which has been validated in Italian and for use in MS [22, 23]. It has 7 questions for anxiety and 7 for depression.

For each question, subjects choose one out of four possible answers with scores ranging from 0 to 3. Possible subscale scores thus range from 0 to 21, both for anxiety and for depression. Based on norms, a score of 0–7 is normal, 8–10 borderline abnormal, and 11–21 abnormal. Anxiety and depression were deemed clinically significant in the presence of subscale scores ≥ 8.^23^

The *Multidimensional Scale of Perceived Social Support (MSPSS*) measures individuals’ perceptions of support received from three sources: family, friends, a significant other [24]. It has a total of 12 items, with four items per subscale (family, friends, significant other). For each item, a higher score indicates a higher level of perceived social support. To calculate the mean subscale scores, the items of each subscale are summed and then divided by number of items of it. The three subscale scores are summed to obtain an overall perceived social support score.

The MSPSS has been validated in Italian and found to show good internal consistency (Crohnbach’s α: from 0.71 to 0.89 for the three subscales), and it has already been used in MS [25, 26]. and in caregivers of people with other diseases [27].

The *Self-Expanded Disability Status Scale (self-EDSS)* measures subjects’ level of disability. It is a descriptive scale derived from the original EDSS [28] and the patient-assessed Patient Determined Disease Steps scale [29]. A recent evaluation of the feasibility and reliability of self-EDSS found the instrument to be acceptable [30].

*Independence in Activities of Daily Living (ADL)*: this scale is designed to assess six basic ADL functions: bathing, dressing, transfers, toileting, feeding and continence [31]. The functions/activities are scored 1 or 0 according to whether or not the subject can perform them. The total score thus ranges from 6 (maximum performance) to 0 (no ability to perform any of the functions/activities).

### Statistical analysis

STATA Statistical Software: Release 17 (StataCorp LP, College Station, TX, USA) was used for the analysis. Descriptive statistics including means, standard deviations, frequencies and percentages were used to analyse the data.

Normality of the primary outcome was tested using the Kolmogorov-Smirnov test, and normal distribution of data was not confirmed. To meet model assumptions of normality, the ZBI total score underwent square root transformation.

Univariate linear regression was used to examine the direction and size of the relationships between caregiver burden and the variables examined, i.e., caregiver characteristics, caregiver resources (internal and external), caregiver-care recipient relationship, intensity of care, and care recipient characteristics. The extent to which the variables explained caregiver burden was analyzed using a hierarchical approach (block-wise analysis). The variables that composed the blocks used in the multivariate analysis were selected by adopting a critical level of significance (*p* ≤ 0.10) in the univariate analysis. Caregiver characteristics were entered in the first block; internal and external caregiver resources in the second block; caregiver-care recipient relationship and intensity of care in the third block; and care recipient characteristics in the last block. Results are presented as coefficients and standard errors. The regression models included the following indicators: R [2], R [2] change (Δ R [2]), *p*-value.

The multiple regression model was checked for multicollinearity: a variable was dropped if the variance inflation factor (VIF) was 10 or over or if the tolerance limit was less than 0.1. Both VIF and tolerance were found to be acceptable in all cases.

## Results

### Caregiver characteristics

200 family caregivers were enrolled in the study. The mean caregiver age was 58.7 years (range 19–85 years); 51.0% of caregivers (*n* = 102) were male and 49.0% (*n* = 98) female; 40.5% (*n* = 81) were working and 63.5% (*n* = 127) had a medium/high level of education. Most caregivers (87.5%, *n* = 175) lived with their care recipient; in 59.0% (*n* = 118) of cases, the caregiver was supported by other people.

At least one disease was reported by 53.0% (*n* = 106) of the caregivers: 28.5% (*n* = 57) had just one condition, 16.0% (*n* = 32) two, and 8.5% (*n* = 17) at least three. Hypertension (28.0%), heart disease (10.0%), and gastric ulcer (8.0%) were the most common conditions, followed by diabetes and depression (each present in 6.5%). The caregivers with at least one disease were older than those who had none (61.8 vs. 55.1 years, *p* = 0.0002).

Mood disorders were reported by 57.0% (*n* = 114) of the caregivers, in particular 51.5% (*n* = 103) reported clinically significant anxiety, and 25.0% (*n* = 50) clinically significant depression (cut-off ≥ 8 on the respective HADS subscales).

Analysis of caregiver knowledge of MS revealed a high percentage of incorrect answers (> 50%) on some items concerning the epidemiology, course and symptoms of the disease.

The caregivers performed a range of tasks: transportation (89.5%), personal assistance such as bathing (59.0%), toileting (42.5%) and dressing (56.5%), provision of emotional (62.5%) and cognitive (64.0%) support, and home and family maintenance (88.0%).

### Internal and external caregiver resources

Among the different coping strategies, the caregivers were more likely to display problem-focused (mean standard score 3.2 (SD 0.6)) and positive attitude-based (mean standard score 3.1 (SD 0.6)) coping, and less likely to adopt a denial strategy (mean standard score 1.4 (SD 0.4)).

In 118 cases (59.0%), caregivers were supported in caring activities by other people such as a formal carer (34.7%) or other family members (65.3%), while 44.0% received domestic help. The MSPSS scores, referring to perceived social support from different sources, were the following: family 17.4 (SD 5.0), significant other 17.2 (SD 4.8), friends 15.0 (SD 5.2).

### Caregiver-care recipient relationship and intensity of care

The caregivers were mainly spouses (69.5%, *n* = 139), while offspring accounted for 11.5% (*n* = 23), parents for 8.5% (*n* = 17), and other relatives for 10.5% (*n* = 21). Around 9.5 (± 7.3) hours per day were devoted to caregiving, this time increasing significantly with increasing disability of the PwMS as measured by EDSS scores (β = 0.97, *p* = 0.005). The caregivers had been looking after their care recipient for an average of 13.4 (± 9.5) years.

### Care recipient characteristics

Of the individuals with MS receiving care, 60.5% were female. The care recipients had a mean age of 58.4 (SD 11.8) years (range: 26–87). Progressive disease courses were the more common type (45.5% secondary progressive and 35.0% primary progressive vs. 19.5% relapsing-remitting) and mean illness duration was 24.7 (SD 11.7) years.

The EDSS score ranged from 0 to 9.0 with a mean value of 6.4 (SD 1.5), and disability levels were distributed as follows: mild (EDSS: 0–3.5) in 6.0%, moderate (EDSS: 4–6.5) in 48.0%, and severe (EDSS: ≥7) in 46.0% of cases.

**Fig. 1:**
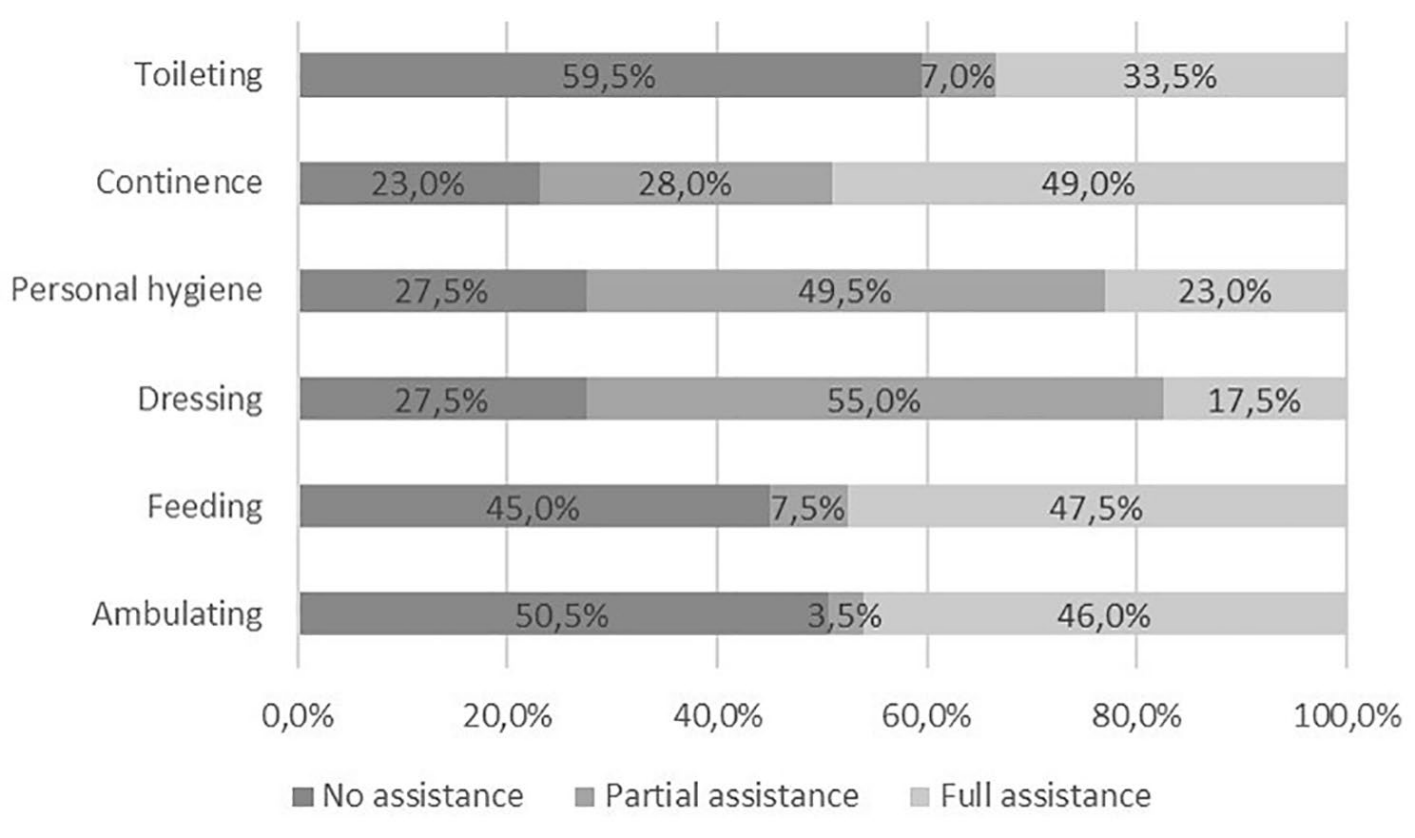
Distribution of care recipients’ status in each ADL item

Analysis of care recipients’ independence, measured using the ADL scale, showed considerable difficulties (need of partial or full assistance) in all items. These data showed, indirectly, the type of assistance provided by caregivers (Fig. 1).

### Caregiver burden

**Fig. 2:**
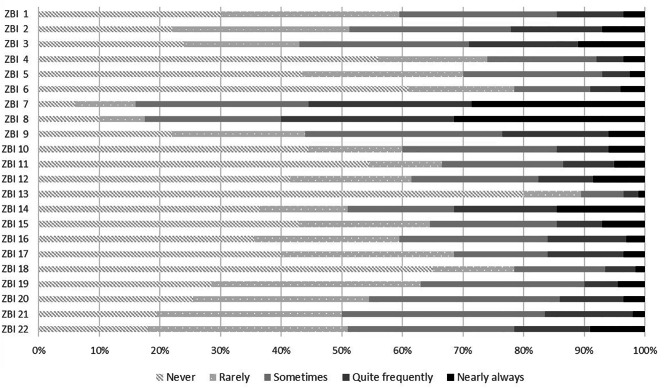
Distribution of caregiver’s answers to each ZBI scale item

As shown by the data collected using the ZBI, approximately one third of the caregivers (32%) did not think that caregiving was affecting their health (ZBI score ≤ 20). Figure 2 shows the distribution of the caregivers’ answers to the single items. The items where caregivers most frequently replied “sometimes”, “quite frequently”, or “nearly always”, i.e., the ones likely contributing most to caregiver burden, were: “Worry about patient’s future” (ZBI 7) 84.0%; “Patient is too dependent” (ZBI 8) 82.5%; “Worry about fulfilling different responsibilities” (ZBI 3) 57.0%, and “Feel could do better” (ZBI 21) 50%.

ZBI short items description: 1 Patient asking for too much help; 2 Not enough time for caregiver; 3 Worry about fulfilling different responsibilities; 4 Embarrassed about patient’s behaviour; 5 Feel angry; 6 Negative effects on other relationships; 7 Worry about patient’s future; 8 Patient is too dependent; 9 Feel strained; 10 Health affected; 11 Inadequate privacy; 12 Social life suffering; 13 Feel uncomfortable having friends visit because of the patient; 14 Expected to be the only caregiver; 15. Financial stress; 16 Feel unable to take care of the patient for much longer; 17 Sense of losing control over life; 18 Wish somebody would take over the care; 19 Feel uncertain of what to do; 20 Feel should do more; 21 Feel could do better; 22 Feel burdened.

### Potential factors influencing caregiver burden

Table 1 shows the results of the univariate analysis. Caregiver burden was associated with caregiver characteristics, namely, the presence of at least one disease (β = 0.78, *p* < 0.001) and levels of anxiety and depression as indicated by the relative HADS subscores (β = 0.20 and β = 0.23, *p* < 0.001, respectively); with internal and external caregiver resources, i.e., respectively, a denial coping strategy (β = 0.12, *p* = 0.023) and poor social support (as indicated by the MSPSS total score) (β=−0.02, *p* = 0.014), and with care recipient variables, i.e., a progressive (primary or secondary) as opposed to relapsing-remitting disease course (β = 1.00, < *p* = 0.001), greater disability (β = 0.37, *p* < 0.001), and less independence (β=−0.23, *p* < 0.001). Caregiver-care recipient relationship and intensity of care were not significantly associated with caregiver burden in the univariate analysis.

**Table 1:** Association between caregiver burden and the study variables

	Univariate linear regression models	
	β (SE)	***P*** value
**Caregiver characteristics**		
Sex (F)	−0.34 (0.22)	0.124
Age	−0.01 (0.01)	0.369
Level of education (Primary school)	1	
High school	−0.17 (0.24)	0.473
University	0.44 (0.32)	0.180
Currently employed (No)	0.24 (0.22)	0.277
Presence of at least one disease (No)	0.78 (0.21)	**< 0.001**
CareKoMS score	0.04 (0.03)	0.143
Anxiety (HADS subscale score)	0.20 (0.02)	**< 0.001**
Depression (HADS subscale score)	0.23 (0.03)	**< 0.001**
**Internal and external caregiver resources**		
Problem focus (COPE subscale score)	0.05 (0.04)	0.233
Positive attitude (COPE subscale score)	−0.01 (0.02)	0.921
Religion (COPE subscale score)	0.01 (0.02)	0.880
Social support (COPE subscale score)	0.01 (0.04)	0.728
Denial strategy (COPE subscale score)	0.12 (0.05)	**0.023**
Social support (MSPSS total score)	−0.02 (0.01)	**0.014**
**Caregiver-care recipient relationship and intensity of care**		
Caregiver-care recipient relationship (partner)	1	1
Parent/child	0.52 (0.27)	0.060
Other parents or friends	−0.46 (0.36)	0.196
Time devoted to care daily (in hours)	0.03 (0.01)	0.077
Duration of care (in years)	−0.01 (0.01)	0.334
**Care recipient characteristics**		
Sex (F)	0.27 (0.22)	0.223
Age	−0.01 (0.01)	0.885
Disease duration (in years)	−0.01 (0.01)	0.307
Disease course (RR)		
SP/PP	1.00 (0.27)	**< 0.001**
Disability level (EDSS score)	0.37 (0.07)	**< 0.001**
Independence level (ADL score)	−0.23 (0.05)	**< 0.001**

β, beta; SE, standard error; CareKoMS: Caregivers’ Knowledge of Multiple Sclerosis; HADS: Hospital Anxiety and Depression Scale; COPE: Coping Orientation to Problems Experienced; MSPSS: Multidimensional Scale of Perceived Social Support; RR = relapsing-remitting, SP/PP = secondary progressive/primary progressive; EDSS: Expanded Disability Status Scale; ADL: Independence in Activities of Daily Living. In brackets reference category

Table 2 shows how the block-wise inclusion of additional covariates improved the fit of the models to the caregiver burden data. The caregiver characteristics included in the first block, i.e., presence of at least one disease, anxiety score, and depression score, were found to be significant predictors of burden. The model explained 40.9% of the observed variance (*F* (3, 196) = 45.28, *p* < 0.001). Internal and external caregiver resources, included in the second block, resulted in no R [2] change (Δ R^2^ = − 0.001, *p* = 1.00) with the model now explaining 40.8% of the observed variance (*F* (3, 193) = 26.62, *p* < 0.001). On adding caregiver-care recipient relationship and intensity of care (third block), a moderate but significant R [2] change was observed (Δ R^2^ = 0.031, *p* = 0.018), with the variables now accounting for 43.9% of the observed variance (*F* (8, 190) = 18.56, *p* < 0.001). Finally, the addition of care recipient characteristics produced an improvement accounting for approximately 6% of the observed variance (Δ R^2^ = 0.062, *p* < 0.001). Together, all the variables in the model explained 50.1% of the observed variance (*F* (11, 187) = 17.04, *p* < 0.001)).

**Table 2:** Hierarchical regression analysis predicting caregivers’ burden

	Block 1	Block 2	Block 3	Block 4
	β (SE), ***p*** value			
**Caregiver characteristics**				
Presence of at least one disease (No)	0.50 (0.17), 0.004	0.48 (0.17), 0.006	0.49 (0.17), 0.005	0.45 (0.16), 0.007
Anxiety (HADS subscale score)	0.14 (0.02), < 0.001	0.13 (0.02), < 0.001	0.13 (0.02), < 0.001	0.12 (0.02), < 0.001
Depression (HADS subscale score)	0.13 (0.03), < 0.001	0.12 (0.03), < 0.001	0.13 (0.03), < 0.001	0.11 (0.03), < 0.001
**Internal and external caregiver resources**				
Denial strategy (COPE subscale score)		0.01 (0.04), 0.757	0.01 (0.04), 0.756	0.06 (0.04), 0.147
Social support (MSPSS total score)		−0.01 (0.01), 0.592	−0.01 (0.01), 0.685	−0.01 (0.01), 0.866
**Caregiver-care recipient relationship and intensity of care**				
Caregiver-care recipient relationship (partner)				
Parent/child			0.50 (0.22), 0.021	0.53 (0.21), 0.012
Other relative or friend			−0.29 (0.29), 0.316	−0.49 (0.28), 0.081
Time devoted to care daily (in hours)			0.02 (0.01), 0.110	0.01 (0.01), 0.878
**Care recipient’ characteristics**				
Disease course (RR)				
SP/PP				0.49 (0.25), 0.050
Disability level (EDSS score)				0.11 (0.08), 0.161
Independence level (ADL score)				−0.08 (0.06), 0.187
R [2]	0.409	0.408	0.439	0.501
Δ R [2], *p* value		−0.001, 1.00	0.031, 0.018	0.062, < 0.001

β, beta; SE, standard error; HADS: Hospital Anxiety and Depression Scale; MSPSS: Multidimensional Scale of Perceived Social Support; COPE: Coping Orientation to Problems Experienced; EDSS: Expanded Disability Status Scale; ADL: Independence in Activities of Daily Living. The reference category is given in brackets; Δ R [2]: R [2] change

Five variables emerged as significant predictors of caregiver burden: the presence of at least one disease (β = 0.45, *p* = 0.007), the anxiety score (β = 0.12, *p* < 0.001), the depression score (β = 0.11, *p* < 0.001), being a parent or child of the care recipient as opposed to their partner (β = 0.53, *p* = 0.012), and caring for a person with a progressive (SP/PP) rather than RR form of MS (β = 0.49, *p* = 0.050).

## Discussion

Due to the growing life expectancy and associated dependence of many people with chronic illnesses and disability, informal carers, such as family caregivers, are increasingly forming an invisible workforce providing them with support and direct care. This study focuses on caregivers of PwMS who are not fully independent (in activities of daily living or in any other way).

For the first time, experiences and needs of Italian MS caregivers have been described besides exploring potential factors influencing their burden. According to the literature, informal caregivers tend to be family members, spouses in particular; [11] they provide a broad spectrum of support, ranging from help in activities of daily living to emotional support [5]. Family caregivers support healthcare systems, not only in Italy but also around the world [7].

While the care recipients in this study included a higher percentage of females, their caregivers were evenly split between males and females. This finding seems to support what is shown in the wider caring literature, namely that women take on the majority of caring responsibilities, a situation that may be linked to gender role expectations [32].

As reported by Manouchehrinia et al., the disability levels and care needs of PwMS tend to increase with age [33]. Since the caregivers in our sample were mainly spouses/partners, and therefore of a similar age to the PwMS, ageing-related health complications were more likely to be an issue for them, too. Caring can be more difficult for individuals with health problems [34], and may therefore increase their care burden.

In our sample, as expected, the caregivers performed a variety of tasks [35]; whereas some of these (such as personal care-related tasks) are straightforward and only to be expected, many others (related to the progression of the disease, for example, or the need to provide emotional support) are not necessarily envisaged initially. Therefore, when these needs arise, caregivers can experience feelings of stress and uncertainty.

Also in agreement with literature findings [36], we observed a high prevalence of mood disorders in our sample, particularly anxiety possibly linked to fears concerning the evolution of the disease and its consequences. Caregivers who are not sure what to expect in terms of the prognosis of the disease, or where to access formal support, in other words, who lack the knowledge necessary to provide adequate care or plan for the future, can feel overwhelmed. In fact, we observed a link between feelings of uncertainty about the future and limited knowledge about MS.

The physical and emotional demands and the time-intensive nature of caring for PwMS often affect the caregiver’s own physical and emotional health. Caregivers have to deal with not only the presence of the disease, but also the unpredictability of its prognosis, and also the fact that it can lead to severe physical and cognitive impairment. Therefore, caregivers, particularly when they are also the life partners of the affected person, may have to face considerable lifestyle and role adjustments that can cause them emotional distress and reduce their quality of life [37].

### Caregiver burden

Approximately two-thirds of the caregivers included in our study (68%) reported a perceived burden of care (ZBI score > 20). Analysis of the single ZBI scale items showed that uncertainty about the future, related to the disease prognosis and progressive worsening of the care recipient’s conditions, could make them feel overwhelmed and unable to provide adequate care. Uncertainty generated by the unpredictability of the disease can make it difficult to make both short- and long-term plans and thus increases the caregiver burden [38].

### Potential factors influencing caregiver burden

The results of the hierarchical regression analysis suggest that caregiver health variables (presence of at least one disease together with anxiety and depression scores) explained much (40.9%) of the observed variance of burden; adding elements related to the carer-care recipient relationship and the intensity of care explained a further 3%, while care recipient characteristics, such as disease course, explained an additional 6%. Internal and external caregiver resources did not contribute significantly.

Our results suggest that physical and mental health are important predictive factors of the care burden. The presence of at least one disease in the caregiver was associated with a higher burden, supporting the suggestion that health problems can make caring more difficult [34]. Furthermore, “as time passes, carer ageing and comorbidities add to the complexity and burden of the disease” [39]. Similarly, psychological problems, such as mood disorders, can be associated with perceived burden in caregivers; indeed, emotional strain associated with caring for PwMS is the most commonly reported challenge, and has strong associations with aspects of mental health, including caregiver depression and anxiety [32]. Since caregivers play a critical role in helping PwMS navigate the disease trajectory, uncertainty over the evolution of the disease, its clinical symptoms, and the associated caring tasks seems to be a key factor in the experience of caregiver burden, producing considerable psychological distress [40].

It is worth underlining our interesting finding regarding the generational relationship between caregiver and care recipient. The perceived care burden was found to be higher in the case of parent-offspring dyads (different generations) compared with ones made up of spouses/partners, which may indicate greater empathy between peers with regard to care needs. Finally, our results confirmed the impact of disease progression on caregiver burden: caregivers of people with primary or secondary progressive forms experienced a greater burden than those caring for people with relapsing-remitting MS [41].

It is important remember that, in view of the study’s cross-sectional design, caution should be exercised when interpreting the relationships between the variables considered. Future studies are needed to further establish our findings.

### Limitations

Given the retrospective and cross-sectional nature of the study, it is difficult to accurately establish the temporal relationship between predictor variables and outcomes. Future longitudinal studies are needed to examine the suggested causal relations. Besides, the scales used in the study were self-reported and this may lead to several biases. Finally, other potential factors that could influence the caregiver burden were not included in the analysis.

## Conclusion

The MS caregivers in this study were mainly spouses, male or female, with mean age of about 59 years. More than half had at least one health condition, and approximately 58% had mood disorders. The caregiving tasks varied and were probably determined by a need to fill gaps resulting from deficits in formal healthcare support. The caregivers were found to spend a large part of their day caring for their care recipient, and to have done so for a long period of time.

As national health and welfare systems struggle to cope with the socioeconomic costs generated by the rise in chronic diseases [42], informal caregivers continue to make up the shortfall, becoming, in the process, both “professionals” in disease management and “hidden patients”. It is important remember that when caregiving becomes a burden, this affects the well-being of both the caregiver and the care recipient. Identifying ways to decrease the burden is therefore important for ensuring the well-being of both parties. Our results highlighted the importance of good physical and mental health in decreasing perceived burden. Working to alleviate psychological distress through mechanisms designed to promote resilience, optimism and self-efficacy in caregivers may be a valid approach. Uncertainty over the future emerged as a recurring theme. In this regard, psychoeducation-based interventions as well as simple measures such as providing MS caregivers with appropriate information might be an effective way of reducing worries and perceived burden.

## Data Availability

The data underlying this article will be shared on reasonable request to the corresponding author.
